# No difference for changes in BMD between two different cementless hip stem designs 2 years after THA

**DOI:** 10.1038/s41598-021-85424-x

**Published:** 2021-03-15

**Authors:** Karen Dyreborg, Søren Solgaard, Michael Skettrup, Michael Mørk Petersen

**Affiliations:** 1 Department of Orthopaedic Surgery 6011, Rigshospitalet, Inge Lehmanns Vej 6, 2100 København Ø, Denmark; 2 Department of Hip and Knee Surgery, Herlev-Gentofte University Hospital, Gentofte Hospitalsvej 8, 2900 Hellerup, Denmark

**Keywords:** Randomized controlled trials, Outcomes research, Surgery

## Abstract

This study evaluates how 2 different total hip arthroplasty (THA) stems compares regarding adaptive bone remodelling. The stems are both proximally porous coated, aiming for proximal fixation, but with different dispersal of the coating. They are also differently designed regarding the distal tip of the stem. We aimed to investigate if there is a difference in periprosthetic adaptive bone remodelling between two different designs. From February 2016 to September 2017, we randomised 62 patients, 1:1 (mean age = 64 years, Female/Male = 28/34), scheduled for an uncemented THA to receive either an EBM or a BM THA stem. We performed dual-energy x-ray absorptiometry (DEXA) scans within a week after surgery and at 3, 6, 12 and 24 months with measurements of bone mineral density (BMD) in the 7 Gruen zones (region of interest (ROI) 1–7). Additionally, Oxford Hip Score and Harris Hip Score were collected at 6, 12 and 24 months. We found a decrease in BMD between the postoperative and the 24-months values in all ROIs for both stems. The greatest decrease over time was seen for both groups in the ROI1 (BM = − 8.4%, p = 0.044, and EBM = − 6.5%, p = 0.001) and ROI7 (BM = − 7%, p = 0.005, and EBM = − 8.6%, p < 0.0005). We found a tendency in ROI2–4 of a higher degree of bone loss in the EBM group. However, this difference only continued beyond 6 months in ROI4 (24 months: BM = − 1.2% and EBM = − 2.8%, p = 0.001). The stems show similar adaptive bone remodelling and are clinically performing well.

## Introduction

Total hip arthroplasty (THA): heralded in 2007 as the operation of the century^1^. From one day to another, patients can walk again, go skiing again and play golf again. THA is a life-changing procedure.

From previous studies we know that the bone mineral density (BMD) (g/cm^2^) decreases directly after surgery in response to the operative trauma and postoperative immobilisation, later on, because of the phenomenon called stress-shielding and finally, in some patients, because of foreign body reaction^2,3^. A decrease in BMD is not beneficial in general but even less so when an uncemented joint replacement is performed. The BMD is proportionally related to the strength of the bone and low levels of BMD lead to increased risk of fracture—a complication known for its unwanted mortality rates^4,5^. Furthermore, the more inferior the bone quality, the more challenging revision surgery becomes.

However, adaptive bone remodelling will begin postoperatively. Adaptive bone remodelling is determined by biomechanical factors, i.e. load transmission, and related to the balance in bone metabolism^6,7^. Other factors which affect the durability of the prosthesis are the design of prostheses, the use of cement or not, and the different inherent biological circumstances in the individual patients.

Previous studies have shown an initial decrease in BMD followed by a slow rise toward baseline values. At 12 months postoperatively stabilisation is seen at a new and either lesser or equal BMD compared to baseline; this phenomenon has been proven most pronounced in the regions corresponding to Gruen zones 1, 4 and 7^8–10^. Some studies also report a slight, but steady decline in BMD beyond 12 months^11,12^.

This study aimed to investigate the regional adaptive bone remodelling between 2 slightly different designs of porous-coated uncemented titanium alloy hip prosthesis; the Bi-Metric Porous Primary (BM) stem and the Echo Bi-Metric Full Proximal Profile (EBM) stem. To do this, we used dual-energy x-ray absorptiometry (DEXA)^13,14^. This evaluation was found relevant because the BM has been on the market for > 30 years with satisfying results; The EBM is the successor of the BM, made with a design based on the BM design but with theoretical enhancements. The EBM was introduced in 2007, and to our knowledge, no randomized controlled trial (RCT) has been made to evaluate this stem. In 2018 784 BM stems were used and 626 EBM stems were used in Denmark (of a total of 7054 cementless THAs; the Corail stem being the most used (3148 in 2018))^15^.

Hypothesis: The uncemented EBM THA stem shows better adaptive bone remodelling compared to the uncemented BM THA stem.

## Methods

In this randomised controlled trial, we enrolled and randomised 62 patients 1:1, planned for an uncemented primary THA to receive either an Echo Bi-Metric Full Proximal Profile (EBM) (n = 31) or a Bi-Metric Porous Primary (BM) (n = 31) THA stem, both from Zimmer Biomet (Warsaw, Indiana).

### Eligibility criteria

The inclusion criteria were: Patients with primary osteoarthritis bound to undergo THA at the Herlev-Gentofte Department of Orthopaedic Surgery, Hellerup, Denmark, age 30–75 years and informed consent.

The exclusion criteria were: Infections, disorders of the bone structure, disorders of the bone metabolism (or use of medications affecting the bone structure or metabolism such as steroids), pregnancy, inability to cooperate, inability to understand, read or speak Danish, and medicine or alcohol abuse.

Secondary exclusion criterion: Technical shortcomings, such as poor image quality.

### Sample size

Our power analysis estimated a minimum of 20 people in each group based on a minimal relevant difference at 7.5% (BMD change between the 2 groups), SD = 8%, Type 1 error = 5% and type 2 error = 15%. This was based upon a clinical estimation and similarity to other studies^16,17^. We included 31 to accommodate future dropouts.

### Randomisation and surgery

The allocation was performed as block randomisation with blocks of ten and was done on the day of surgery using a closed, non-transparent envelope opened in the operation theatre, when the patient was ready for surgery. The allocation was confirmed by all relevant staff (2 nurses, the surgeon and the primary investigator).

Before the first operation, the randomisation code was generated by a web-based program, and the envelopes with each allocation were sequentially numbered and kept securely stored. The sequence was locked away at a different location (Rigshospitalet). A colleague outside the project did the randomisation allocation sequencing and packaging of envelopes. Screening and enrolment were done by the primary investigator.

All operations were performed by 1 of 4 experienced hip surgeons. Surgery was performed according to standard procedure with a posterolateral approach and following the manufacturers’ recommendations. There were no differences in preparation of the femurs for the 2 different stems. Before each procedure and allocation, the surgeons templated both stems on the calibrated radiographs to anticipate the size and position of the stem and cup.

All patients received a 32 mm chrome-cobalt head and an Exceed ABT ringloc-x acetabular shell with a highly cross-linked polyethylene liner. Surgery was performed under spinal or general anaesthesia. Physiotherapy began on the day of surgery, and the patients were mobilised with full weight-bearing using crutches. All patients were given oral anticoagulants according to the praxis of the department, and prophylactic antibiotics during the first 24 h. Postoperatively, patients were given Paracetamol and, if needed, Oxycodone Hydrochloride. No NSAIDs or steroids were used. Due to visual differences of the 2 prostheses, the surgeon and health personnel were not blinded. The participants were all blinded.

### Implants

**Figure 1:**
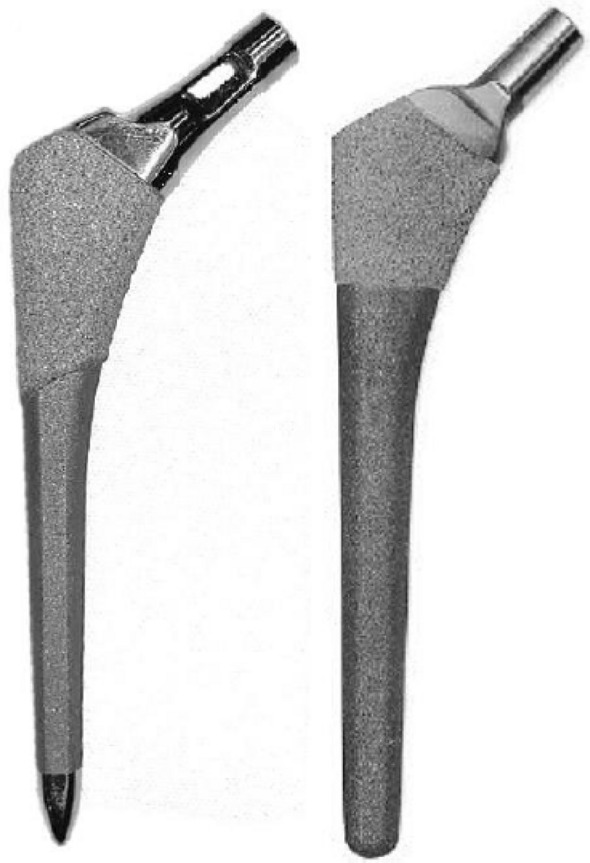
The stems. The Echo Bi-Metric stem (EBM) to the left and the Bi-Metric stem (BM) to the right.

Both stems are press-fit titanium alloy stems with a proximal plasma spray porous titanium coating designed for biological fixation. The distal part of the stems has a roughened titanium surface for bone on-growth. The BM was introduced in 1984 and has shown good clinical results and excellent stem survival in register studies^18–21^. The EBM was introduced in 2007 and is–compared to the BM–a relatively new implant that is now in routine clinical use. The stem uses many of the features of the BM while integrating new design features to enhance the clinical performance further: reduced neck dimensions (designed to allow for increased range of motion and decreased risk of neck impingement); a polished neck (designed to reduce debris should impingement occur); a polished bullet-shaped distal tip (designed to reduce distal stresses). Besides, the proximal porous coating has been extended further to support the biological fixation (Fig. 1).

### Dual-energy x-ray absorptiometry

DEXA-scans were performed at the Department of Orthopaedic Surgery, Rigshospitalet, Copenhagen, Denmark. Scans were done within a week of surgery and at 3, 6, 12 and 24 months after surgery. We scanned the operated hip, from the level of the acetabulum to a minimum of 2 cm distal to the tip of the stem, and both proximal tibiae (as to compare with a region outside the hip region, which has not been operated on, but has the same relative load transfer). Sandbags secured correct rotation of the leg. A Norland XR-46 bone densitometer (Norland Corp, Fort Atkinson, WI) was used for measurements of BMD (g/cm^2^). It has customised software with an adjustable threshold for metal exclusion, allowing BMD measurements of the bone adjacent to the implants (this is called “high-density point exclusion”). Scan speed was set at 45 mm/s and the pixel size at 1.0 × 1.0 mm. Quality control of the machine was performed using daily calibration before the first scan. All the DEXA-scans were carried out by trained health professionals.

**Figure 2:**
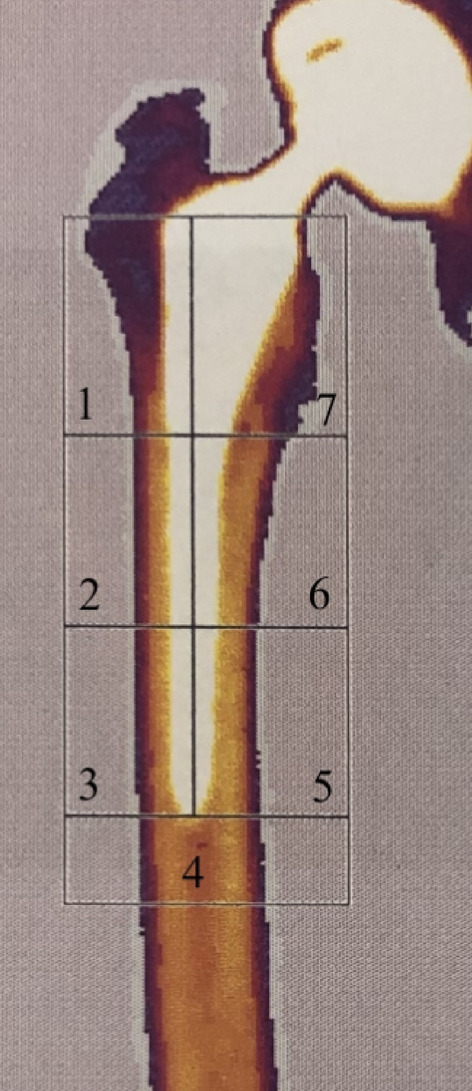
Regions of interest (ROIs) of the periprosthetic BMD measurements.

When the 24-months-scans were all performed, 7 regions of interest (ROI) were placed manually on the femur scans to represent the 7 Gruen zones as in an anteroposterior X-ray (Fig. 2). The high-density point exclusion was kept at the same level for the individual patient for the entire follow-up, but varied from 3500 to 6000 between patients. We used modified Gruen zones, as described by ten Broeke et al.^13^. All ROI-markings were performed starting with the marking of ROI3, then ROI2, ROI1, ROI4, ROI5, ROI6 and ROI7.

DEXA scans of both the proximal tibiae were made for comparison with bone metabolism in other parts of the body. On these scans, a single ROI was placed just distal to the subchondral plates of the tibial condyles.

In all these 9 separate regions the local BMD was automatically calculated by the software. All the ROIs were placed after all scans were made, to ensure that they were exactly alike for the individual participant from one data-point to another. Equally, all ROIs were placed for one participant at a time and solely by the primary investigator.

Precision of the BMD measurements was evaluated by double measurements of 11 participants by calculating the coefficient of variation (CV = (standard deviation (SD) / mean) × 100%). For the different ROIs we found the following CV presented as mean and range: CV_ROI1_ = 2.6% (0.1–9.5%); CV_ROI2_ = 0.9% (0–3.2%); CV_ROI3_ = 1.6% (0.2–3.2%); CV_ROI4_ = 1.3% (0.1–2%); CV_ROI5_ = 1.4% (0–3.7%); CV_ROI6_ = 1.7% (0.2–4.3%); CV_ROI7_ = 3.3% (0.4–8.1%); CV_ROIknee_ = 2.2% (0.1–12.1%).

### Outcomes

#### Primary outcome measure

The percentage change of mean bone mineral density in the 7 Gruen zones at 24 months (set against the first scan) with a comparison between the BM and the EBM group.

#### Secondary outcome measures

Adaptive bone remodelling measured by the change of bone mineral density in the proximal tibiae bilaterally at 24 months (compared with the first scan).

Clinical outcome monitored with Harris Hip Score (HHS) and Oxford Hip Score (OHS)^22–25^ preoperatively and at 6, 12 and 24 months. HHS is an objective outcome measure and has a maximum score of 100 points (as the best possible outcome) covering pain, function, absence of deformity, and range of motion. The OHS is a subjective outcome measure to assess pain, functional ability and daily activities. It produces overall scores running from 0 to 48, with 48 being the best outcome possible. Minimally important difference estimate for HHS is 18.0^26^, and for OHS it is 5^24^.

### Ethics and registration

The study was approved by the local Ethical Committee (H-4-2014-079), by the Danish Data Protection Agency (GEH-2015–079, I-Suite no. 03764) and prospectively registered at ClinicalTrials.gov (NCT02656771, 15/01/2016). The study was carried out in accordance with the principles of the Helsinki declaration.

All patients were informed orally and in writing as prescribed in the recommendations and requirements from the local Scientific Committees. Informed consent was obtained from all subjects.

### Statistics

Data were tested for normality by histograms, QQ-plots and Kolmogorov–Smirnov, and found normally distributed.

For the evaluation of potential differences between the mean BMD changes of each group, we used an independent t test. A t test for paired data was performed for comparison of the immediate postoperative BMD value and the value after 24 months. Furthermore, repeated measurements ANOVA was made for evaluation of within-group changes in BMD over time.

The Mann Whitney U test was used to compare the groups with regards to clinical scores.

All data are presented as mean with SD or 95% confidence intervals (CI_95_) unless otherwise reported. The statistical software SPSS version 24 (IBM Corp. New York, USA) was used.

## Results

**Figure 3:**
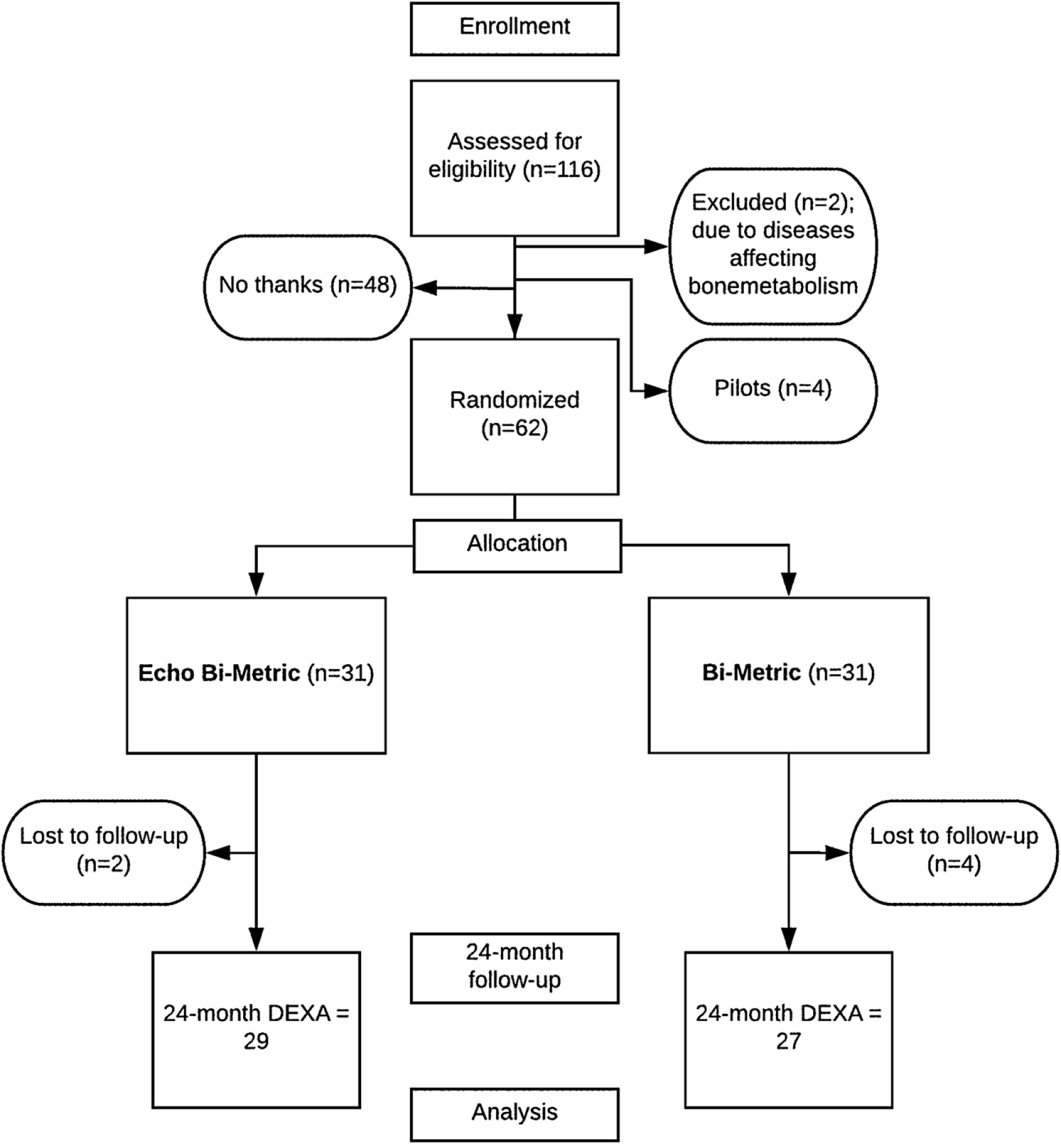
Flowchart.

Of 116 people assessed for eligibility, 66 were included, 4 as pilot-patients and consequently, 62 were randomised (Fig. 3) (mean age = 64(range 49–74) years, Female/Male = 28/34). In the BM group, 2 participants died due to other reasons before 24 months had passed; 2 participants were reoperated, 1 due to periprosthetic fracture and 1 due to excessive subsidence, both within 3 months of the primary operation.

In the EBM group, 1 participant did not want to proceed in the study beyond 12 months, and in 1 case we had to exclude the patient from the study at 24 months because of technical difficulties. Data for these patients are included in the study for as long as they individually have participated. The groups were similar with respect to patient characteristics and perioperative data (Table 1).

**Table 1:** Baseline demographics.

	Bi-Metric	SD (range)	Echo bi-metric	SD (range)
Age (mean)	66	6 (49–74)	63	8 (50–74)
Sex (male/female)	17/14		17/14	
Height (m)	1.77	0.09 (1.6–1.96)	1.76	0.09 (1.6–1.9)
Weight (kg)	84	19 (50–124)	83	17 (54–122)
BMI (kg/m^2^)	26.6	4.9 (17.9–38.3)	26.7	3.4 (20.1–35.6)
Operated side (right/left)	18/13		15/16	
Cup size (mean)	55	3 (50–62)	56	3 (50–62)

### Primary outcome

At 24 months we saw the most marked decrease in BMD in both groups in the ROI1 (BM = − 8.4%, p = 0.04, and EBM = − 6.5%, p = 0.001) and ROI7 (BM = − 7%, p = 0.005, and EBM = − 8.6%, p < 0.0005) followed by ROI2 (BM = − 4%, p = 0.007, and EBM = − 5.2%, p < 0.0005) (Table 2). In the remaining ROIs, there were just minor fluctuations. Statistically significant differences between the immediate postoperative BMD and the 24-months BMD values were found in most ROIs for both stems.

**Table 2:** Periprosthetic bone mineral density (BMD).

BMD	Postoperative		3 months		6 months		12 months		24 months		0–24 months t test p value		ANOVA p value	
	EBM (n = 30)	BM (n = 31)	EBM (n = 30)	BM (n = 30)	EBM (n = 30)	BM (n = 28)	EBM (n = 30)	BM (n = 28)	EBM (n = 29)	BM (n = 27)	EBM	BM	EBM	BM
**ROI1**														
Mean SD	1.214 (0.17)	1.219 (0.15)	1.186 (0.18)	1.252 (0.19)	1.16 (0.2)	1.225 (0.24)	1.14 (0.17)	1.195 (0.29)	1.122 (0.15)	1.125 (0.24)	0.001	0.044	0.609	0.546
ΔBMD% CI_95_			− 1.1 (− 3.6; 1.7)	− 0.16 (− 0.1; 5.8)	− 3.8 (− 1.2; 8.2)	− 0.16 (− 7.9; 6.1)	− 5.6 (− 8.6; − 2)	− 2 (− 7.5; 8.3)	− 6.5 (− 9.7; − 2.9)	− 8.4 (− 16.4; − 1.7)				
p value			0.094		0.348		0.461		0.659					
**ROI2**														
Mean SD	1.993 (0.28)	1.935 (0.19)	1.916 (0.28)	1.915 (0.21)	1.925 (0.29)	1.886 (0.21)	1.934 (0.3)	1.884 (0.23)	1.884 (0.3)	1.847 (0.23)	< 0.0005	0.007	0.725	0.587
ΔBMD% CI_95_			− 3 (− 4.3; − 1.5)	− 0.24 (− 1.7; 1.3)	− 3.4 (− 5.4; − 1.6)	− 1.4 (− 3.2; 0.5)	− 3.1 (− 5.1; − 1.2)	− 2 (− 3.9; − 0.05)	− 5.2 (− 7.1; − 3.2)	− 4 (− 6.7; − 1.5)				
p value			0.014		0.129		0.408		0.459					
**ROI3**														
Mean SD	2.117 (0.29)	2.180 (0.26)	2.019 (0.29)	2.128 (0.25)	2.046 (0.28)	2.107 (0.24)	2.072 (0.29)	2.116 (0.25)	2.046 (0.28)	2.094 (0.23)	< 0.0005	< 0.005	0.771	0.063
ΔBMD% CI_95_			− 4 (− 5.3; − 2.8)	− 1.7 (− 2.7; − 0.7)	− 3.4 (− 4.; − 2.5)	− 2 (− 3; − 0.9)	− 2.3 (− 3.3; − 1.4)	− 1.7 (− 2.8; − 0.4)	− 3 (− 4.2; − 2)	− 2.3 (− 3.3; − 1.3)				
p value			0.01		0.059		0.46							
**ROI4**														
Mean SD	1.943 (0.29)	2.003 (0.31)	1.88 (0.28)	1.948 (0.3)	1.877 (0.29)	1.956 (0.29)	1.9 (0.31)	1.984 (0.3)	1.888 (0.31)	1.985 (0.31)	< 0.0005	0.2	0.729	0.629
ΔBMD% CI_95_			− 3.2 (− 4.3; − 2.3)	− 1.5 (− 3; − 0.1)	− 3.4 (− 4.7; − 2.4)	− 0.7 (− 2; 0.8)	− 2.5 (− 3.9; − 1.3)	0.8 (− 1.4; 3.1)	− 2.8 (− 4.3; − 1.6)	− 1.2 (− 0.6; 3)				
p value			0.053		0.009		0.021		0.001					
**ROI5**														
Mean SD	2.118 (0.27)	2.167 (0.26)	2.059 (0.28)	2.129 (0.26)	2.083 (0.28)	2.126 (0.25)	2.116 (0.29)	2.133 (0.26)	2.104 (0.3)	2.134 (0.23)	0.306	0.857	0.762	0.097
ΔBMD% CI_95_			− 2.3 (− 3.6; − 1.2)	− 0.8 (− 2.1; 0.4)	− 1.6 (− 3.1; − 0.3)	− 0.5 (− 1.5; 0.6)	− 0.1 (− 1.8; 1.5)	0.06 (− 1.3; 1.3)	− 0.85 (− 2.5; 0.5)	0.19 (− 1.9; 1.9)				
p value			0.119		0.229		0.915		0.375					
**ROI6**														
Mean SD	1.943 (0.25)	1.924 (0.21)	1.88 (0.26)	1.846 (0.24)	1.887 (0.26)	1.861 (0.22)	1.901 (0.27)	1.844 (0.22)	1.907 (0.26)	1.855 (0.24)	0.015	0.042	0.750	0.702
ΔBMD% CI_95_			− 2.7 (− 4.2; − 1.5)	− 3.7 (− 5.9; − 1.7)	− 3 (− 4.2; − 1.8)	− 3 (− 4.1; − 1.7)	− 2.1 (− 3.6; − 0.8)	− 4.1 (− 5.6; − 2.5)	− 1.7 (− 3; − 0.3)	− 3.3 (− 5.7; − 0.9)				
p value			0.464		0.973		0.057		0.273					
**ROI7**														
Mean SD	1.675 (0.21)	1.791 (0.23)	1.542 (0.2)	1.686 (0.26)	1.515 (0.22)	1.671 (0.26)	1.498 (0.22)	1.605 (0.25)	1.517 (0.22)	1.61 (0.26)	< 0.0005	0.005	0.732	0.464
ΔBMD% CI_95_			− 6.8 (− 8.8; − 5)	− 1.5 (− 7.3; 9.3)	− 9 (− 11.1; − 6.8)	− 4 (− 9.3; 6.4)	− 10.1 (− 12.3; − 7.9)	− 7.7 (− 13.5; 2.3)	− 8.6 (− 11.3; − 5.8)	− 7 (− 12.9; 3.9)				
p value			0.37		0.364		0.577		0.711					

BMD in the 7 Gruen zones from postoperatively to 24 months after surgery.

When comparing the BMD changes over time between the 2 different stems, we found a tendency in ROI2–4 towards different early bone remodelling patterns, within the first 6 months, with a higher degree of bone loss in the EMB group (with p values between 0.01 and 0.13). However, this difference in bone remodelling pattern only continued beyond 6 months in ROI4, where it was statistically significant 24 months postoperatively (p = 0.001).

Over time, using repeated measures ANOVA, we could not identify any statistically significant changes in BMD in any of the ROI for any of the groups.

### Secondary outcomes

We divided the knee DEXA scans into the tibia on the operated side and the tibia on the not-operated side (Table 3). Initially, there was an increase in the BMD in the EMB and BM group on the operated side (BM = 4.7% and EBM = 1.2%, p = 0.08) and a minor decrease in both groups on the not-operated side (BM = − 0.02% and EBM = − 0.6%, p = 0.7). At 24 months, no significant differences were found between the groups, but a decrease in both groups in both tibiae, with a slightly more substantial loss of BMD in the EBM group was seen (BM = − 1.4% on the operated side and − 5.1% on the not-operated side. EBM = − 5.4% on the operated side and − 7.3% on the not-operated side).

**Table 3:** Bone mineral density (BMD) in the tibiae from postoperatively to 24 months after surgery.

BMD	Postoperative		3 months		6 months		12 months		24 months		0–24 months t test p value		ANOVA p value	
	EBM (n = 30)	BM (n = 31)	EBM (n = 30)	BM (n = 30)	EBM (n = 30)	BM (n = 28)	EBM (n = 30)	BM (n = 28)	EBM (n = 29)	BM (n = 27)	EBM	BM	EBM	BM
**ROI tibia on the operated side**														
Mean SD	0.798 (0.18)	0.837 (0.23)	0.804 (0.19)	0.849 (0.2)	0.787 (0.19)	0.847 (0.22)	0.772 (0.17)	0.820 (0.22)	0.75 (0.18)	0.804 (0.2)	< 0.0005	0.078	0.581	0.304
ΔBMD% CI_95_			1.2 (− 0.8; 3.1)	4.7 (1.6; 8.2)	− 1.2 (− 4.3; 1.9)	0.4 (− 1.6; 2.2)	− 2.8 (− 5.3; − 0.4)	− 1.5 (− 4.8; 1.2)	− 5.4 (− 8.3; − 2.8)	− 1.4 (− 4.9; 1.9)				
p value			0.079		0.384		0.503		0.079					
**ROI tibia the not operated side**														
Mean SD	0.84 (0.17)	0.893 (0.22)	0.834 (0.18)	0.876 (0.21)	0.806 (0.18)	0.89 (0.23)	0.797 (0.18)	0.846 (0.21)	0.777 (0.18)	0.832 (0.21)	< 0.0005	0.001	0.746	0.588
ΔBMD% CI_95_			− 0.6 (− 3.2; 1.9)	− 0.02 (− 2.1; 2.2)	− 4.3 (− 6.6; − 2.2)	− 0.2 (− 2.2; 1.7)	− 5.3 (− 8.1; − 3.1)	− 4.2 (− 6.2; − 2)	− 7.3 (− 10.5; − 4.1)	− 5.1 (− 8.2; − 2.2)				
p value			0.733		0.011		0.5		0.327					

In both groups, we found an increase in HHS and OHS from the preoperative scores to the 24-months-scores as seen in Table 4, and we found no significant differences between the groups.

**Table 4:** Clinical scores. Harris Hip Score (HHS) (maximum score is 100) and Oxford Hip Score (OHS) (maximum score is 48).

Mean (range)	HHS			OHS		
	BM	EBM	p value	BM	EMB	p value
Preoperative	61 (38–82)	67 (31–85)	0.147	24 (10–35)	23 (9–40)	0.667
6 months	92 (61–100)	91 (70–100)	0.221	43 (29–48)	44 (27–48)	0.490
12 months	97 (81–100)	95 (73–100)	0.959	46 (31–48)	44 (26–48)	0.029
24 months	99 (91–11)	98 (49–100)	0.601	47 (43–48)	46 (21–48)	0.901

There were no unintended harms or effects besides the reported two reoperations.

## Discussion

We hypothesized that the uncemented EBM THA stem would show better adaptive bone remodelling compared to the uncemented BM THA stem. Our main findings were that the 2 prostheses have almost similar early-term patterns of adaptive bone remodelling in all ROIs except in ROI4.

The regions ROI1 and ROI7, with most trauma during surgery, most cancellous bone and with the highest risk of stress-shielding, had the lowest levels of BMD. For both stems, we saw a slight decrease in BMD in ROI1 initially and a further decline in the period 3–12 months. In ROI7 the decline was even more substantial. We believe this is caused by stress-shielding in the area, consistent with current knowledge and theory^27^.

Several factors may influence the change in BMD in the femur after THA, either physiological or iatrogenic. In ROI4 the BM group always seemed to have a better adaptive bone remodelling. Maybe this could be explained by the differences of the tip-design, i.e. the “polished bullet-shaped distal tip”-design of the EBM, designed to reduce the distal stress because of less bone ongrowth in the region. There have been speculations regarding the effects of sex and age on BMD in different ROIs, but there seems only to be a consensus that stem design and fixation are the leading influencers on periprosthetic bone remodelling, especially in ROI4 and ROI7^28–30^. It could be proposed that the design of the EBM is not advantageous for adaptive bone remodelling in ROI4. However, the differences found were very small, and we do not find any clinical evidence to back this up. Of course, ROI4 is a critical area, since later periprosthetic fractures might occur just distal to the tip of the prosthesis where the relationship between low BMD and the principle of torque or lever represents a dangerous combination. However, there is also a generally higher loss of BMD in the EBM group (represented by the distal tibiae scans) indicating that other factors contribute to the BMD loss (e.g. age, genetics, movement and so on).

The lower BMD values in ROI4 may be explained by another feature, shared by the 2 prostheses; the proximal weight-bearing design, which could reduce the load distally, with a decreased osteoblast stimulus and hence a lower BMD. The EBM and the BM are both designed for proximal bone ingrowth, theoretically resulting in less calcar and trochanteric stress-shielding and consequently less bone loss.

Compared to other studies of adaptive bone remodelling after THA we found similar values of the BMD and similar patterns of the BMD over time. In 2008 Van der Wal et al.^31^ found a 24 months BMD% difference ranging from − 14.5% in ROI7 and − 11.3% in ROI1 to 2.8% in ROI6, correspondent our findings of BMD differences being greatest in ROI1 and ROI7.

Venesmaa et al. reported in 2003^32^ a general decrease in all ROIs until 6 months and mostly in the calcar region. After 6 months, they reported only minor changes (follow-up was 60 months). This is in line with our results, but in general, we see a decrease over the 24 months.

When comparing the BMD over time in the periprosthetic hip regions with the tibiae BMD over time, we can conclude that there is a general drop in BMD from the postoperative measurements to the 24 months measurements. This emphasises that the explanation of a decreasing BMD over time lays not just in the THA, but it is also a matter of other systemic factors—such as progression of age. Interestingly, we observe an early increase in BMD in the tibiae on the operated side, suggesting a return to normal after a period of immobilisation before the operation.

Limitations to be mentioned are the rather small sample size and the selection of participants.

Even though we included 11(35.5%) persons more than calculated by the power calculation, it is not a large cohort and a non-inferiority/equality study would be interesting. Still, this requires an even larger cohort and is a slow process for a single hospital. We find the above to be the optimal solution when weighing time, investment and results.

Another limitation is our blinding-regimen in which only the patients were truly blinded; not the surgeon, the nurses or the physiotherapists, and not the primary investigator.

In conclusion, both the BM and the EBM stems are clinically performing well and show comparable adaptive bone remodelling. We did not find major differences between the 2 study groups; but, long term follow-up is necessary and is currently being conducted. Our hypothesis is thereby rejected.

We look forward to reporting the 5-year-results for this cohort.

### Data availability

The datasets generated and analysed during the current study are not publicly available since this was not a part of the informed consent signed by the participants, but are available from the corresponding author on reasonable request.
